# Continued Sensitivity of *Plasmodium falciparum* to Artemisinin in Guyana, With Absence of Kelch Propeller Domain Mutant Alleles

**DOI:** 10.1093/ofid/ofw185

**Published:** 2016-08-30

**Authors:** Reyaud Rahman, Maria Jesus Sanchez Martin, Shamdeo Persaud, Nicolas Ceron, Dwayne Kellman, Lise Musset, Keith H. Carter, Pascal Ringwald

**Affiliations:** 1 Ministry of Public Health; 2 Pan American Health Organization, Georgetown, Guyana; 3 Laboratoire de Parasitologie, Institut Pasteur de la Guyane, Cayenne, French Guiana; 4 Pan American Health Organization, Washington, District of Columbia; 5 World Health Organization, Geneva, Switzerland

**Keywords:** artemisinin, C580Y, Kelch propeller domain, malaria, *Plasmodium falciparum*

## Abstract

Because of concerns about possible emergence of artemisinin resistance strains of *Plasmodium falciparum* in mining areas of the interior of Guyana, a 7-day artesunate trial was conducted from March to December 2014. The day-3 parasite clearance rate, the efficacy of artesunate at day 28, and polymorphism of Kelch 13 (*PfK13*)—the marker of artemisinin resistance—were assessed. The study confirmed the continued sensitivity of *P falciparum* to artemisinin. A 7-day course of artesunate was 100% efficacious with only 2% (95% confidence interval, .1%–10.9%) of enrolled subjects positive at day 3. All day-0 parasite samples were wild type. Continued resistance monitoring is nevertheless recommended, given the widespread availability and uncontrolled use of artemisinin drugs in mining areas of Guyana.

Over the past 15 years, artemisinin-based combination therapies (ACTs) have become the first-line treatment for *Plasmodium falciparum* malaria in almost all malaria-endemic countries. In conjunction with long-lasting insecticidal bed nets, the use of these drugs has played a major part in the dramatic reduction in the global burden of malaria observed since 2000 [1]. However, the emergence of artemisinin resistance threatens to undermine these gains.

Artemisinin resistance has been detected in 5 countries in the Greater Mekong Subregion ([GMS] Cambodia, Lao People's Democratic Republic, Myanmar, Thailand, and Vietnam). Artemisinin resistance is characterized by delayed parasite clearance after treatment with an artemisinin monotherapy, or ACT [2]. For ACTs, resistance to the artemisinin derivatives may not result in clinical failures if parasites remain susceptible to the partner drug. In recent studies, mutations in the Kelch 13 (*PfK13*) propeller region have been identified as a molecular marker of artemisinin resistance, being associated with delayed parasite clearance both in vitro and in vivo [3, 4]. In the GMS, the mutations C580Y, R539T, Y493H, and I543T are most frequently associated with artemisinin resistance.

The World Health Organization (WHO) recommends efficacy monitoring for first-line and second-line ACTs every 2 to 3 years in all endemic countries. Such studies aim to determine (1) the proportion of malaria cases with persistent parasitemia at day 3 after treatment with an ACT and (2) the rate of treatment failures at days 28 or 42. In the case of suspected artemisinin resistance (high prevalence of patients with day 3 positive blood smears or emergence of *PfK13* mutations), a confirmatory study using artesunate monotherapy should be conducted, complemented by sequencing of the *PfK13* gene of the parasite samples in patients enrolled on day 0 as recommended by the Technical Expert Group on Drug Efficacy and Response (http://www.who.int/malaria/mpac/mar2016/en/).

Recent studies have raised concerns regarding the possible emergence of artemisinin resistance in the Guiana shield region of South America. In Suriname, a study conducted in 2011 reported that 15 of 48 (31%) evaluable patients treated with artemether-lumefantrine were parasitemic on day 3, compared with 2% in 2005/2006, although therapeutic efficacy was 100% at day 28 and *PfK13* sequencing showed only wild-type isolates [5]. A review of this study demonstrated several methodologic problems (in particular microscopy quality control), raising questions about the results. In 2014, Guyana reported 12 354 cases with 42% of the cases due to *P falciparum* and 58% due to *Plasmodium vivax*. The most malarious regions in Guyana are gold mining areas in the country's interior where a broad range of artemisinin drugs is available in local shops and pharmacies and self-treatment is common. Sequencing of 98 samples from Guyana collected in 2010 found 4 isolates from patients who reported travels to region 7 (Cuyuni-Mazaruni) and one who traveled to region 1 (Barima-Waini) of Guyana that carried the C580Y mutant allele in the *PfK13* propeller domain but with flanking microsatellites different from those observed in Southeast Asia [6].

The primary objective of this study was to assess the possible emergence of artemisinin resistance in Guyana by determining the day-3 parasite clearance rate in patients with uncomplicated *P falciparum* malaria treated with a 7-day course of artesunate monotherapy. Samples from the enrolled patients were also analyzed to assess the association of *PfK13* polymorphisms with clinical response.

## METHODS

This was a one-arm prospective survey conducted from March to December 2014 in patients recruited in the Malaria Clinic at the Georgetown Public Hospital Corporation, Georgetown, Guyana. Patients from all over the country can attend the Malaria Clinic, but the majority comes from gold mining areas in the country's interior. All subjects provided written informed consent. Ethical approval was provided by the Ministry of Health in Guyana, after review by its Institutional Review Board (no. 158). The study is registered at The Ministry of Public Health of Guyana (http://www.health.gov.gy/moph/), ACTRN12616000526471.

Eligible subjects were those attending the clinic with microscopically confirmed, uncomplicated *P falciparum* monoinfection (parasitemia of 250–200 000/µL asexual forms) and were >2 years old and able to swallow oral medication. Exclusion criteria were severe malaria, severe malnutrition, febrile condition other than malaria, known drug hypersensitivity, and pregnancy or breast-feeding. A pregnancy test was required for all women of childbearing age.

To facilitate follow up, enrolled patients were housed in a hotel near the Malaria Clinic for days 0–7 and received directly observed treatment with artesunate (4 mg/kg body weight) once daily for 7 days followed by a single dose of primaquine (0.5 mg/kg) on day 8, with out-patient follow up to day 28. Artesunate was provided as 50-mg tablets (Guilin Pharmaceutical, Guangxi, China) and primaquine as 15-mg tablets (Vario Pharma Private Limited, Mumbai, India). Artesunate was quality controlled by North-West University in South Africa. Any patient vomiting within 30 minutes of dosing was retreated; repeat vomiting triggered study withdrawal and rescue treatment with injectable quinine 3 × 10 mg salt/kg per day. Failures were treated with artemether-lumefantrine.

A complete medical history and demographic information were taken at enrollment. All patients received a clinical assessment at enrollment, daily on days 1–7 and on days 14, 21, and 28. Adverse events were recorded throughout the study.

Duplicate Giemsa-stained thick and thin blood films were obtained at the time of initial screening (day 0), every 8 hours for 7 days after the initial dose of artesunate, and on days 14, 21, and 28. Parasite densities were determined as per WHO procedures by 2 independent microscopists [7]. Parasite densities were calculated by averaging the 2 counts; blood smears with discordant results for the presence of parasites were re-examined by a third senior expert microscopist for final decision. The study was monitored twice by an independent consultant, and slides were quality controlled by an expert microscopist of the Malaria Branch, Centers for Disease Control and Prevention (Atlanta, GA) who confirmed the parasite clearance results after internal quality control and WHO curation of the data.

Therapeutic efficacy was evaluated as per WHO guidelines using adequate clinical and parasitological response (ACPR), ie, absence of parasitemia without previous treatment failure [7]. To differentiate a recrudescence from a newly acquired infection, blood samples for parasite polymerase chain reaction (PCR) genotyping were collected on Whatman 3MM filter paper on day 0 and day of failure. In cases of treatment failure, paired filter papers were used for parasite deoxyribonucleic acid extraction and genotyping, based on WHO methods [8]. Artemisinin resistance of *P falciparum* strains was also explored by examining polymorphisms in the *PfK13* propeller domain at day 0 using a nested PCR protocol followed by Sanger sequencing using primers specific for *P falciparum*. The amplicon used for sequencing covered 656 base pairs (amino acids 49–705), which included the K13 propeller domain as recommended by WHO [4].

The required study sample size of 35 patients was calculated based on an estimated prevalence of parasitemic patients at day 3 after artesunate therapy >10% at a confidence level of 95%. Adding 20% to allow for study withdrawals gave a target sample size of 42 patients.

Study efficacy outcomes were the proportion of patients' who still had parasites in their blood smears on day 3 and ACPR at day 28. Parasite clearance time and slope half-life were calculated using the WHO parasite clearance estimator. Safety outcomes were the incidence of adverse events. Data analysis was performed using a Microsoft Excel spreadsheet provided by WHO. Both are available at http://www.who.int/malaria/areas/drug_resistance/en/.

## RESULTS

Fifty patients were enrolled from June to November 2014. All enrolled patients complied fully with the study procedures and protocols. There were 3 withdrawals, 2 on day 21 and 1 on day 28; all were triggered by *P vivax* infection. Most patients were male (41 of 50) and over 15 years of age (49 of 50). Mean age was 30.6 years (range, 12–58 years), mean weight was 66.7 kg (range, 35.8–106 kg), mean temperature was 37.2°C (range, 36.6–39.9°C), and geometric mean parasitemia was 6281 µL (range, 359–155 100). The patients came from 3 of the 4 regions in Guyana where malaria remains endemic: region 1 (Barima-Waini) (26%), region 7 (Cuyuni-Mazaruni) (54%), and region 8 (Potaro-Siparuni) (16%). One patient had been living in region 9, and 1 patient originated from Venezuela. Parasitemia at day 3 was observed in 1 of 49 patients (2%; 95% confidence interval [CI], .1–10.9) with a median parasite clearance time of 38.5 hours (range, 8–80 hours). This patient did not have any outstanding feature that differed from the other patients. Median slope half-life (range) evaluated in 45 patients was 2.97 hours (1.09–5.9 hours), with 2 patients only presenting with a slope half-life >5 hours. In 47 evaluable patients, ACPR at day 28 was 100% (95% CI, 92.5–100). Because there were no recrudescences, PCR adjustment was not required. All 50 patients enrolled in the study, including the patient presenting with parasites at day 3, had day-0 *P falciparum* samples that were *PfK13* wild type in the propeller domain. No serious adverse events were reported in the study. Two mutations were observed outside the propeller region: D80G in all the samples and K189T in 35.7% of them.

## DISCUSSION

Guyana adopted artemether-lumefantrine plus primaquine as the first-line treatment for *P falciparum* in 2004. Efficacy of this combination was high after implementation and no delayed clearance was reported at that time. There are no recent therapeutic efficacy data for Guyana, as a trial conducted with artemether-lumefantrine in 2011–2012 failed to meet quality-control requirements [2].

The Guiana shield region is an area of high population mobility due to mining activities where uncontrolled use of antimalarial drugs, including many artemisinin drugs, is widespread. Concern regarding the emergence of artemisinin resistance justified further investigation at the country level. This study conducted in 2014 by the malaria control program in Guyana did not confirm the presence of artemisinin resistance. None of the patients had delayed parasite clearance, and all parasites were wild type in the propeller domain of the *PfK13* gene.

Several reasons could explain the discrepancy between this 2014 clinical study and the detection of *PfK13* mutants in parasites collected in 2010 [6]. The objective of the current study was not to confirm the existence of *PfK13* mutants in regions 7 and 8, because the results of the 2010 survey reporting C580Y mutants in very low frequency (5 samples of 98) were published after the 2014 clinical study was terminated. Although 70% of the patients included in the 2014 study were infected in regions 7 and 8, the probability of finding a C580Y mutant during this clinical trial including 50 patients only was very low. The working definition of suspected artemisinin resistance set by WHO considers that a ≥5% prevalence of a single *PfK13* mutant signifies selection of the genotype in the parasite population, which requires further investigation. Clonal expansion under drug pressure in the presence of a mobile migrant population attracted by mining activities would have most probably led to an increase in the prevalence of mutants and regional spread over the last 5 years. So far, no C580Y mutants have been reported in neighboring countries [9]. Most likely, parasites carrying the C580Y mutations reported in Guyana in 2010 have not expanded or gone to fixation but could have spontaneously disappeared due to a reduced fitness cost, the high efficacy of ACTs used in the region, or absence of background mutations [10]. Although the epidemiological context of malaria is different, similar emergence of *PfK13* mutants without clonal expansion and spread has been reported in Africa and seems to be related to natural polymorphism of the *PfK13* gene in a parasite population rich in low-frequency alleles [11]. Finally, we do not think that inclusion of patients with previous antimalarial drug intake has modified the results of efficacy or *PfK13* mutants. Previous antimalarial treatment is a well known factor that selects for resistant parasites for both artemisinin and partner drug [12]. An additional survey focusing specifically on regions 7 and 8 is needed to follow up on the trend of prevalence of *PfK13* mutation in Guyana.

## CONCLUSIONS

In conclusion, this study did not confirm the presence of artemisinin resistance in Guyana, but continuous surveillance is necessary.
